# Ophthalmological manifestations of hereditary transthyretin amyloidosis

**DOI:** 10.5935/0004-2749.20220099

**Published:** 2025-08-22

**Authors:** Francisco de Assis Aquino Gondim, Joana Gurgel Holanda Filha, Manoel Odorico Moraes Filho

**Affiliations:** 1 Drug Research and Development Center & Department of Internal Medicine, Neurology Division, Universidade Federal do Ceará, CE, Brazil; 2 Drug Research and Development Center & Department of Surgery, Universidade Federal do Ceará, CE, Brazil; 3 Drug Research and Development Center & Department of Physiology and Pharmacology, Universidade Federal do Ceará, CE, Brazil

**Keywords:** Cataract, Amyloidosis, familial, Glaucoma, Neuropathy, Transthyretin, Catarata, Amiloidose familiar, Glaucoma, Neuropatia, Transtirretina

## Abstract

Transthyretin familial amyloidosis is the most common form of inherited systemic amyloidosis worldwide. The condition develops secondary to more than 100 different point mutations in the transthyretin gene (18q12.1). The mutations lead to abnormal amyloid deposits, mainly in the heart and peripheral nerves. Leptomeningeal and mainly ocular involvement is common. Although there are several different types of treatment available, ocular involvement, which occurs also in liver transplant recipients, remains a major challenge, progressing even in liver transplant recipients. Patients with ocular involvement require efficient ophthalmological follow-up to prevent vision loss. In this review, different forms of ocular involvement characterizing the subtypes of transthyretin mutations were described, and the effects of different treatments were summarized. Further research is necessary to fully elucidate these issues.

## Abstract (pt)

A amiloidose familiar por transtirretina é a forma mais comum de amiloidose sistêmica hereditária mundialmente. A condição é secundária a mais de 100 mutações ponto diferentes no gene da transtirretina (18q12.1). Mutações levam a depósitos anormais de amilóide principalmente no coração e nos nervos periféricos. O envolvimento leptomeníngeo, e principalmente ocular também é comum. Apesar do advento de várias formas diferentes de tratamento, o envolvimento ocular ainda é um grande problema, progredindo mesmo em pacientes submetidos à transplante hepático. Nesta revisão, avaliamos as diferentes formas de envolvimento ocular presentes entre os subtipos de mutações da transtirretina e os efeitos dos diferentes tratamentos disponíveis. O envolvimento ocular é desafiador na amiloidose familiar por transtirretina, exigindo acompanhamento oftalmológico eficiente para mitigar a perda de visão. Mais pesquisas são necessárias para auxiliar na resolução dessas questões.

## INTRODUCTION

The German botanist Mathias Schleiden (1804-1881) coined the term “amyloid” in 1839 to describe plant products similar to starch, colored blue by iodine, and found in the layers of the primary cell membrane^(1)^. The term derives from the Latin word *amylum* for starch. Together with Theodor Schwann and Rudolf Virchow, Schleiden is known for the unified cell theory of all living organisms, namely, that all living organisms (plants and animals) are composed of cells^(1)^. Virchow was the first to describe amyloid in the brain (*corpora amylacea*) and use the word in the animal literature, although Nicolaus Fontanus may have described amyloid deposits in the liver and spleen centuries earlier (1639)^(1)^. Virchow mistakenly believed that amyloid was similar to starch or cellulose. Friedreich and Kekulé described the protein nature of amyloid and absence of carbohydrate (high nitrogen content), thus shifting the direction of research to consider amyloid as an abnormal protein resulting from fibril formation and conformational changes^(2)^.

The term “amyloidoisis” is used to describe a group of diseases characterized by systemic or localized amyloid deposits due to a wide range of medical conditions, including malignancies, inflammatory diseases, as well as hereditary and familial disorders. The use of Congo red and thioflavin staining improved diagnostic accuracy and allowed a better understanding of the amyloid structure. Despite their heterogeneity, all forms of amyloid have a fibrillar ultrastructure, and this feature has been considered as the second criterion for the definition of amyloid^(2)^.

**Figure 1:**
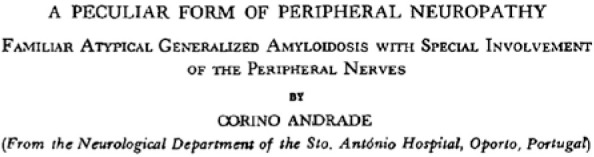
Front page of the manuscript published by Corino de Andrade: Brain. 1952;75:408-427. Permission obtained from Oxford University Press, license number 4863390399804 from 7/21/2020.

In 1939, Corino de Andrade, a Portuguese neurologist, documented a new form of idiopathic peripheral neuropathy. He received training in pathology in France (under the supervision of Jean Alexandre Barré) and Germany (Max Planck Institute). With such a background, he was able to identify the presence of amyloid deposits in nerve biopsies. In 1952, he reported his findings from 74 patients followed for 10 years in a seminal paper published in *Brain*^(3)^. He described 4 characteristic findings of hereditary transthyretin-related (ATTRv) amyloidosis: 1) paresis in the extremities (mainly the legs); 2) early impairment of pain and temperature; 3) gastrointestinal disorders; and 4) sexual and sphincter disorders (Figure 1). In this review, we focus on the ophthalmological findings of this particular type of hereditary amyloidosis.

## METHODS

We conducted a review of the main clinical studies investigating the different subtypes of transthyretin (TTR) point mutations as well as correlations of the clinical, neurological, and ophthalmological findings with the disease course and treatment used. We also briefly reviewed some basic epidemiological and etiological factors as well as mechanisms underlying different ophthalmological manifestations. Studies were selected using an advanced PubMed search and keywords such as “familial amyloid polyneuropathy”, “eye”, ophthalmological complications/manifestations”, and “transthyretin”. We also searched for specific disease subtypes (entities and manifestations) seen in patients with TTR mutations, such as glaucoma, vitreous opacities, scalloped iris, and amyloid angiopathy. All studies with an abstract and a full text in Portuguese, English, French, Spanish, and German were initially screened, and the most relevant references were selected (up to a maximum of 100 references allowed by *Arquivos Brasileiros de Oftalmologia*). Medical websites listing the different TTR mutations and phenotypical correlations were searched to facilitate phenotypic differentiation.

## RESULTS

### Epidemiology

Initially, the occurrence of ATTRv amyloidosis was found to be limited to former Portugal, Swedish, and Portuguese colonies (e.g. Brazil) as well as a few other countries considered to be Portuguese commerce partners (e.g. Japan, Cyprus), where the Val30Met mutation is more prevalent. However, later it became clear that this type of amyloidosis was far more widespread. Especially in Portugal, patients with Val30Met mutations could develop amyloidosis early or late in life (earlyand lateonset disease). However, for reasons still unclear, the same mutation affected only older patients in nonendemic areas^(4)^. In 2018, the prevalence of ATTRv amyloidosis was estimated to be between 5526 and 38468 individuals worldwide^(5)^. However, subsequent reports from other countries suggested a far greater number of cases, especially considering the forms with predominant cardiac involvement. Several mutations have a more restricted geographic distribution and a particular set of phenotypic manifestations (Table 1). The prevalence of different eye diseases also vary according to the mutation and geographic distribution, as described in greater detail below and in table 1.

**Table 1:** List of transthyretin mutations associated with ocular findings with respective ethnic background and associated systemic findings

Mutation		Origin	Neurological/Systemic ∆s	Ophthalmological findings	References	
**Exon 2** Cys10Arg (p.Cys30Arg)		Hungary/USA	PN, H	VO	^77^	
Asp18Glu (p.Asp38Glu)		USA	PN, H	VO, tortuous retinal vessels	^29,78^	
Val30Gly (p.Val50Gly)		USA	PN, LM, seizures	VO, glaucoma, bilateral intermediate uveitis	^11,16^	
Val30Met (p.Val50Met)		Por, Japan, Swe	Classic, predominant PN	Full spectrum: KCS, pupillary ∆s, OAG, VO, conjunctival/retinal angiopathy	^26,61,76^	
Phe33Ile (p.Phe53Ile)		USA, J	PN	VO	^29,36^	
Phe33Val (p.Phe53Val)		C, UK	PN	VO	^79^	
Phe33Cys (p.Phe53Cys)		Poland, USA	PN, H, K	VO (including as the first manifestation of FAP)	^80^	
Arg34Gly (p.Arg54Gly)		Kosovo	PN, CTS	VO: pseudopodia lentis; neovascular glaucoma	^72^	
Lys35Thr (p.Lys55Thr)		C, J	PN, CTS, H	VO81		
Ala36Pro (p.Ala56Pro)		C, Gr, I, J, USA	OMV, PN, deafness, strokes	VO, retinal vasculitis, VH, early ocular disease	^55,75,82^	
Trp41Leu (p.Trp61Leu)		Russia, USA	Skin disease, PN	VO as the first symptom; posterior subcapsular cataract	^83^	
Gly47Arg (p.Gly67Arg)		USA, Italy	PN, CTS, H, LM	VO	^29^	
**Exon 3** Thr49Ala (p.Thr69Ala)		Italy, France	PN, H	VO: pseudopodia lentis	^84^	
Ser50Arg (p.Ser70Arg)		Japan, Mexico	PN, H	Dry eyes in 18%	^85^	
Gly53Ala (p.Gly73Ala)		UK	PN, H, K, LM, OM	VO, retinal involvement	^86^	
	Glu54Gly (p.Glu74Gli)	UK	PN, H	VO, spontaneous subconjunctival hemorrhages, retinal angiopathy	^29,87^	
	Glu54Lys (p.Glu74Lys)	Japan, Turkey	PN, H	VO	^88^	
	Leu55Arg (p.Leu75Arg)	C, G	PN, LM	VO	^81^	
	Leu55Gln (p.Leu75Gln)	Spain, Swe	PN, H, CTS	Glaucoma, VO, cataracts	^89^	
	Leu55Pro (p.Leu75Arg)	C, D, G, USA	Aggressive early PH, H	KCS, INO, lacrimal gland amyloid deposits, pupillary ∆s, VO	^90^	
	Leu58Arg (p.Leu78Arg)	Japan	PN, H, CTS	VO	^91^	
	Phe64Ser (p.Phe84Ser)	Canada (I), UK	LM, H, CNS, PN, deafness	VO, blindness, Horner syndrome	^92^	
	Gly67Arg (p.Gly87Arg)	Bangladesh	PN	KCS, VO (“glass-wool”), conjunctival angiopathy	^53^	
	Lys70Glu (p.Lys90Glu)	Finland	PN, CTS	VO, secondary glaucoma, cataracts	^93^	
	Val71Ala (p.Val191Ala)	France, Spain	PN, H, K	VO, retinal hemorrhages and angiopathy	^54^	
	Ser77Tyr (p.Ser97Tyr)	France, G, USA	PN, H, K	Conjunctival lymphangiectasia	^65^	
	Gly83Arg (p.Gly103Arg)	C (Han)	PN, H	VO (isolated initial presentation of FAP), glaucoma, xerophthalmia, dyscoria	^94,95^	
Ile84Ser (p.Ile104Ser)		Hungary, S, USA	PN, H	VO	^96^	
Glu89Lys (p.Glu109Lys)		Japan	PN, CTS, H	KCS, VO, retinal microangiopathy, VH	^66^	
**Exon 4** Ile107Val (p.Ile127Val)		Brazil, Japan, USA	PN, H	KCS	^42^	
Tyr114Cys (p.Tyr134Cys)		Japan, USA	PN, LM, H, myopathy	Pupillary ∆s, secondary glaucoma, KCS, VH, VO, conjunctival angiopathy, CRVO	^58,69,72^	

C= China; CRVO= central retinal vein occlusion; CTS= carpal tunnel syndrome; D= Dutch; G= Germany; FAP= Familial amyloid polyneuropathy; Gr= Greece; H= heart; KCS= keratoconjunctivitis sicca; I= Italian; INO= internuclear ophthalmoplegia; J= Jewish; K= kidney disease; LM= leptomeningeal involvement; OAG= open-angle glaucoma; OMV= oculomeningovascular; PN= peripheral neuropathy; Por= Portugal; S= Swiss; Swe= Sweden; UK= United Kingdom; USA= United States; VH= vitreous hemorrhages; VO= vitreous opacities.

### Overview of disease mechanisms

TTR, also known as prealbumin, is a protein transporter for thyroxine and retinol-binding protein. It is mainly synthetized by the liver. TTR is the only protein produced by the choroid plexus; therefore, it is present in cerebrospinal fluid and serum. It is also produced by the retinal and ciliary pigment epithelium^(6)^. TTR is formed by 4 identical subunits (tetramers) with a molecular weight of 55 kDa. Each subunit consists of 127 amino acids arranged in 8 antiparallel β sheets (A-H). Moreover, the subunit contains a central channel with 2 thyroxine binding sites, of which only one is occupied in physiological conditions. The mature protein is formed after cleavage from a sequence of 20 amino acids. Thus, there are discrepancies in the nomenclature depending on whether the translation of the initial amino acid or after the cleavage of 20 amino acids is considered. For example, the most common mutation is Val30Met using the old nomenclature, but it is p.ATTRVal50Met when considering the cleavage of the initial sequence of 20 amino acids.

TTR mutations cause molecular instability and protein misfolding. Amyloid fibrils formed by TTR aggregate in the extracellular space. The morphology of the fibrils is type of mutation-dependent. In early-onset disease, fibrils are thick and long, while in late-onset forms, they are usually short and thin^(7)^. Subsequently, due to impaired blood-nerve barrier, the protein deposits accumulate in the nerves and in other organs depending on the mutation, mainly the kidneys, heart, eyes, meninges, and brain^(6)^.

### Neurological findings

As mentioned above, ATTRv amyloidosis due to the Val30Met mutation was first described as a polyneuropathy, with the initial involvement of small (type C) nerve fibers leading to sensory (pain, numbness, and paresthesias) and early autonomic impairment, including erectile dysfunction, diarrhea, constipation (or alternating diarrhea and constipation), and orthostatic hypotension^(3)^. The clinical progression of disease was followed by motor impairment resulting in emaciation, ultimately leading to death within 15 to 20 years after symptom onset. In Portugal, it became clear that in addition to this early and more slowly progressing disease, there was another form characterized by late-onset and faster progression (death within 10-15 years). In Japan, late-onset ATTRv Val30Met amyloidosis from nonendemic areas was associated with the time from disease onset to death of approximately 7.3 years^(8)^. Another very common characteristic is the presence of carpal tunnel syndrome^(3)^.

Less common forms of neuropathy include phenotypes marked by predominant motor involvement, including fasciculations, mimicking amyotrophic lateral sclerosis, seen in Japan (mutation Ile127Val)^(8)^. The disea se can also mimic chronic inflammatory demyelinating polyneuropathy, leading to inappropriate treatment with immunosuppression. Another important phenotype is asymmetric neuropathy simulating mononeuritis multiplex, leading to the wrong diagnosis of leprosy. The diagnosis can be challenging when the neuropathy phenotype overlaps with other forms of axonal cryptogenic neuropathy or when the patient has additional risk factors for neuropathy, such as diabetes, vitamin B_12_ deficiency, or history of alcohol abuse^(8)^. An additional confounding factor is the diagnosis of lumbar spinal stenosis, which can significantly delay the diagnosis in elderly patients^(9)^. Although spinal stenosis is an important differential diagnosis, a link between TTR mutations and spinal stenosis has also been revealed, with amyloid deposits contributing to lumbar spinal stenosis in a patient with genetically confirmed ATTRv^(9)^.

TTR mutations can also result in central nervous system involvement. Cardiac involvement with heart failure and arrhythmias may lead to ischemic and hemorrhagic stroke that can progress to vascular dementia^(6,9,11)^.

### Cardiac and additional non-ophthalmological findings

With disease progression, ATTRv amyloidosis causes a wide range of cardiac manifestations, including restrictive cardiomyopathy and cardiac insufficiency with progressive conduction disorders^(12)^. These conditions can eventually lead to sudden cardiac death due to arrhythmia as well as stroke^(13,14)^. Importantly, some mutations cause predominant cardiac involvement, with minimal or not clinically significant neuropathic involvement^(14)^. The most common mutations associated with predominant cardiac involvement are Ser77Tyr, Thr60Ala, Val122Ile, and Gly89Gln^(6,15)^. The patterns of cardiac involvement and cardiac work-up were reviewed elsewhere^(15)^.

The extent of involvement in ATTRv amyloidosis can be far greater, especially in advanced stages and in specific subtypes of mutations. Cases with oculoleptomeningeal^(16)^ and renal involvement were described^(17)^. Oculoleptomeningeal amyloidosis is more commonly associated with central nervous system disease, including dementia. Ocular involvement in this type of ATTRv amyloidosis is discussed below. Nephropathy is more frequent in patients with late-onset neuropathy, and microalbuminuria can be the first manifestation of ATTRv amyloidosis in patients without neuropathy symptoms^(17)^. Kidney disease can also significantly contribute to mortality in this population^(17)^.

### Ophthalmological findings

#### Historical introduction

**Figure 2:**
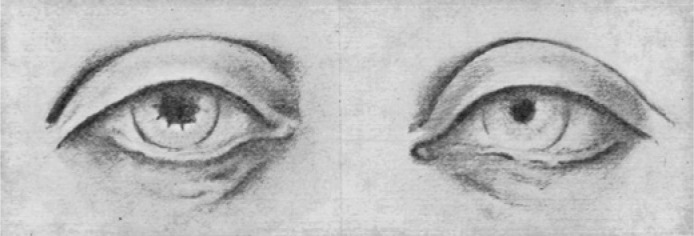
Original figure from the manuscript published by Corino de Andrade (Brain 1952;75:408-427) showing anisocoria with irregular outlines and fringed edges (scalloped pupils). Permission obtained.

In his seminal paper, Corino de Andrade^(3)^. included a figure depicting pupillary abnormalities (Figure 2). He described peculiar pupillary appearance with irregular outlines and fringed edges, as well as impaired direct and consensual light response^(3)^. However, the term “scalloped pupils”, currently used to describe abnormalities typical for ATTRv amyloidosis, was coined in 1975 by Lessell et al.^(18)^.

Historically, De Navasquez and Treble^(19)^ provided the first description of generalized amyloid disease with nervous system and ocular involvement, although it was not clear whether this was hereditary or secondary amyloidosis. However, the patient’s age (36 years) and disease course were consistent with ATTRv amyloidosis. In addition to marked autonomic involvement with diarrhea, impotence, and muscle wasting, they described pupillary abnormalities: small, regular, and central pupils that did not react to light and reacted only sluggishly on convergence. One year after the paper by Corino de Andrade, Kantarjian and Dejong published a more detailed report of eye involvement^(20)^.

They described 2 sisters with severe peripheral neuropathy as well as gastrointestinal, endocrine, and ocular symptoms. The patients showed progressive vision loss, bilateral exophthalmos with marked proptosis (they also suffered from thyroid disease), extensive vitreous opacities, patches of exudate along the retinal vessels, unequal dilated pupils that reacted to accommodation but not to light. One of the sisters died. Death occurred also in the father, who exhibited similar symptoms (bilateral exophthalmos and unreactive pupils to light)^(20)^. A few years later, Falls et al. reviewed 6 cases of ATTRv amyloidosis with ocular involvement, including the 3 patients reported by Kantarjian and Dejong, but with a greater focus on ophthalmological manifestations^(21)^. They stated that ocular involvement is common in ATTRv amyloidosis and affects at least 8% of patients. They emphasized the possibility of retinal hemorrhages as well as amyloid deposition in the walls of the central retinal artery and short posterior ciliary vessels^(21)^. In addition to a detailed description of vitreous opacities, glaucoma was reported in one-third of the patients. Subsequently, Kaufman^(22)^ described open-angle glaucoma and probably one of the first reported attempts to treat vitreous opacities with aspiration and transplantation in patients with ATTRv amyloidosis^(22)^. The occurrence of glaucoma in a 25-year-old patient pointed to the role of ATTRv amyloidosis in the pathogenesis of this condition. Lid ecchymosis and “glass-wool” vitreous opacities were proposed as ocular signs suggesting the correct diagnosis^(22)^. Kaufman was also the first to describe the amyloid content in vitreous opacities^(22)^. Only decades later, amyloid deposits were isolated from the vitreous by using a technique previously applied to differentiate subtypes of amyloidosis in urinary sediments^(23)^. Vallat et al. evaluated vitreous amyloid deposits from a 35-year-old man and provided an electron microscopic description of vitreous body sediments with the fibrillar ultrastructural pattern of amyloid^(23)^.

#### Epidemiology

In patients with ATTRv amyloidosis, ophthalmological findings vary depending on the specific mutation. As previously mentioned, the first reports revealed the prevalence of ophthalmological findings in about 8% of cases^(21)^. However, the most recent literature describes eye involvement in 10% to 24.1% of patients with ATTRv amyloidosis^(24)^. In most cases, clinically relevant ocular involvement develops late, but eye disease was also reported as the first manifestation of ATTRv amyloidosis^(25)^.

To date, the largest study with an ophthalmological evaluation of ATTRv amyloidosis was conducted in 513 carriers of the Val30Met mutation from Portugal^(26)^. Unfortunately, the study was biased in that 72% of clinically affected patients had already undergone liver transplant. Eye involvement was asymmetrical, and no ocular abnormalities were observed in asymptomatic patients (7%). Tear break-up time and Schirmer test abnormalities were the most common findings (79.5% and 67%, respectively), followed by amyloid deposition in the iris (38.4%) and in the anterior capsule of the lens (32.9%), scalloped iris (27.9%), glaucoma (20%), vitreous amyloidosis (17.4%), abnormal conjunctival vessels (14%), and retinal amyloid angiopathy (4%). Abnormal Schirmer test results, scalloped iris, and vitreous amyloidosis were more common in older patients. Transplant recipients had more extensive amyloid deposition in the iris, anterior capsule of the lens, and vitreous than nontransplant patients. Glaucoma was frequently associated with scalloped iris, while retinal amyloid angiopathy, with vitreous amyloidosis. The first manifestations of ocular involvement after 5 years of disease were abnormal Schirmer and tear break-up time test results^(26)^. Retinal amyloid angiopathy slowly developed after 10 years. In 2 Japanese series, by Kimura et al. (n=49)^(27)^ and Ando et al. (n=37)^(28)^, abnormal conjunctival vessels were slightly more prevalent than keratoconjunctivitis sicca. Kimura et al. reported vitreous opacity in 35%, amyloid deposition along the pupil in 31%, and glaucoma in 20% of patients^(27)^. In contrast, Reynolds et al. reported eye disease in only 24% of patients (mostly women), including vitreous amyloidosis in 100%, glaucoma in 19%, tortuous retinal vessels in 15%, and neurotrophic keratitis in 8% of patients^(29)^.

Didactically, eye involvement in ATTRv amyloidosis can be divided according to mechanisms: 1) direct and secondary effects of sensory and autonomic neuropathy; 2) direct and secondary effects of systemic and ocular TTR deposition; and 3) vascular ophthalmological lesions due to altered TTR production. Several relevant reviews about eye involvement in ATTRv amyloidosis have been published before^(24,30-35)^.

### Disorders predominantly due to sensory and autonomic Neuropathy

#### 1. Decreased tear production (keratoconjunctivitis sicca) and Neurothrophic Keratopathy

Dry eyes are the most common ophthalmological manifestation of ATTRv amyloidosis^(26)^, demonstrated by abnormal tear break-up time and Schirmer test results in up to 80% of the patients. It is also the earliest manifestation that frequently remains undiagnosed until significant impairment^(36)^. Dry eyes leading to keratoconjunctivitis sicca are also the most frequent eye symptom in early ATTRv amyloidosis^(36)^. The largest series to date documented a higher prevalence of dry eyes in all disea se stages, linking abnormal Schirmer and tearing break-up time test results to a combination of aging, amyloid deposits, and neuropathy^(26)^.

Alacrimia due to autonomic neuropathy has been documented in several conditions, such as Allgrove syndrome^(37)^. In our literature review, dry eyes were not generally considered to result from autonomic neuropathy in ATTRv amyloidosis. Most publications attributed keratoconjunctivitis sicca to sensory impairment^(26,38)^. Other authors suggested TTR deposits in the lachrymal glands as the causative factor^(39)^. However, TTR deposits were mainly described in advanced stages^(40)^. Since keratoconjunctivitis sicca can be found early in the disease course, amyloid deposition cannot be the main contributor to the sicca syndrome in ATTRv amyloidosis. It is the most important complication of decreased tear production, observed mainly in women^(28)^. The socalled neurotrophic neuropathy has been attributed to trigeminal nerve involvement^(41)^. We recently reported a case of a patient with ATTRv amyloidosis due to the Ile127Val mutation with repeated corneal ulcers treated by 2 corneal transplants, leading to complete vision loss in one eye^(42)^.

Those conditions are treated with general therapy for dry eyes: artificial tears, eyelid hygiene, and ocular occlusion devices. In one study, 5 patients with severe dry eye disease (refractory to treatment with artificial tears and lacrimal plugs) were successfully treated with cyclosporine eyedrops (0.05%) after liver transplant, with no side effects^(43)^. However, the authors highlighted the risk of dysautonomia of the lacrimal gland and accessory lacrimal glands (meibomian gland dysfunction). The exact mechanisms underlying the effect of cyclosporine in the treatment of keratoconjunctivitis are unknown, but they may include reduced inflammation and corneal damage, restored integrity of the corneal epithelium, and reduced nerve damage^(44)^.

#### 2. Pupillary abnormalities

Corino de Andrade was the first to report pupillary abnormalities in ATTRv amyloidosis^(3)^. Anisocoria was common in his series, with preserved response to atropine and lack of response to pilocarpine. The term “scalloped pupils” was coined by Lessell et al., who considered the condition to result from parasympathetic (ciliary nerves) or sphincter muscle involvement^(18)^. Okajima et al. reported mainly sympathetic postganglionic involvement and additional preganglionic sympathetic disease but were unable to detect parasympathetic disturbances^(38)^.

Rubinow and Cohen reported scalloped pupils in 17 of 24 United States patients with ATTRv amyloidosis and a diverse genetic background^(45)^. None of them had vitreous opacities or corneal amyloid deposition. In Japan, Kimura et al. reported scalloped pupils in 8% of the study group (8 eyes from 5 patients)^(27)^. Ando et al. identified pupillary abnormalities in 43.2% of patients: decreased light reflex in 23.2%, deformity in 21.6%, amyloid deposition at the pupillary border in 18.9%, and a decrease in light and near reflex in 2.7%^(28)^. The prevalence of pupillary involvement reached 81% during follow-up. Houlden et al. evaluated pupillary abnormalities in hereditary neuropathies^(46)^. They described common pupillary involvement in ATTRv amyloidosis, half of cases with bilateral Horner syndrome and 12.5% of cases with tonic pupils. Ala60 (Irish ancestry) was the most common mutation. Two patients with the Val30Met mutation had normal pupils. Thus, amyloid deposits are less important than autonomic involvement even for scalloped pupils^(47)^. Glaucoma was also reported as a contributing factor to ATTRv amyloidosis^(26,27,33)^.

### Vitreous amyloidosis and eye transthyretin deposition

Soon after ATTRv amyloidosis was first described, ophthalmologists noticed that amyloid deposits caused vitreous opacities^(22)^. TTR was identified in the corneal endothelium, lens capsule, iris epithelium, retinal pigment epithelium, ciliary pigment epithelium, and retinal nerve fibers, except in the lens and tears^(26,32,48,49)^. TTR was shown to bind retinol to retinol-binding protein and to be present at higher levels in the retina and vitreous^(32)^. Since TTR is produced by the retinal pigment epithelium and liver, it was not clear whether its deposits in the eye originated entirely from the pigment epithelium or from systemic deposition or even from wild-type amyloid. Mutant TTR Val30Met cannot cross the blood-eye barrier^(50)^. However, persistent eye disease after liver transplant suggests the retinal pigment epithelium as the main source^(26)^. TTR levels are decreased in patients with diabetes and hypertension, while they are increased in those with leukemia and carcinoma^(32)^. TTR can also be locally produced by the choroid plexus, causing leptomeningeal disease^(51)^.

Vitreous opacities in ATTRv amyloidosis are usually bilateral and asymmetrical^(35,52)^. Their incidence ranges from 5.4% to 35% depending on the study^(35)^. They can be seen in patients with late-onset neuropathy, leptomeningeal and central nervous system involvement, or, more rarely, in those with ophthalmological involvement alone^(24)^. Vitreous amyloidosis is an important differential diagnosis of uveitis^(53)^.

Patients usually report blurred vision and floaters, with a variable degree of painless vision loss progressing from months to years and dependent on amyloid deposition density^(53,54)^. Fundoscopy reveals yellowish-white glass-wool vitreous opacity consistent with amyloid deposits^(53,55)^. Some authors reported that vitreous opacities are the most common ophthalmological change in late-onset Val30Met ATTRv amyloidosis with at least 4 types of deposits: pseudopodia lentis, fibrils, spherical opacities, and prevascular opacities^(36)^. The typical appearance of vitreous amyloid was described as sheetlike, film-like, band-like, cobweb-like, glass-wool-like, cotton-like, and stringy-fibril like^(35)^. In 4 Portuguese patients with Val30Met mutations and vitreous amyloidosis, blurred vision was the first symptom of ATTRv amyloidosis and preceded neurological findings in 2 patients^(56)^. Patients had amyloid deposits in the vitreous with lacy arrangement and attachments to the posterior lens by means of footplates (pseudopodia lentis) and a perifoveal gray ring seen on fundoscopy. Ecography revealed posterior vitreous detachment^(56)^. Pseudopodia lentis (foot plates) are opacities on the posterior lens capsule that can be traced further through clear vitreous into the gray-white meshwork of opacified vitreous^(57)^. In a family with the Phe33Ile TTR mutation (p.Phe53Ile), it was found in 90% of cases^(35)^. The degree of vitreous opacities was graded into 3 stages by Koga et al.^(58)^ In the posterior lens ocular coherence tomography, pseudopodia lentis can appear as solar eruptions^(59)^.

Vitrectomy is one of the best treatments that is frequently required (if associated with visual impairment) to remove amyloid from the vitreous cavity^(60)^. In a series by Venkatesh et al., all patients had intraoperative hyaloid adhesion and glass-wool vitreous^(35)^. Glaucoma was reported in only 1 patient, although it was not caused by amyloid deposit. During a short follow-up, none of the patients experienced recurrence. Amyloid deposits may recur due to residual retrolental vitreous or, most likely, due to ongoing amyloid production by the retinal pigment epithelium^(61)^. Although vitrectomy is highly effective, recurrence can be treated with repeated vitrectomy, anti-vascular endothelial growth factor agents, and panretinal photocoagulation; however, these treatments may be ineffective^(35)^. In a series by Reynolds^(35)^, vitrectomy led to improvement (2 patients had recurrent symptoms), but its long-term effects were not assessed. Monteiro et al. reported better outcomes of vitrectomy in younger patients^(56)^, although 1 patient required another vitrectomy after 41 months, 1 patient developed cataract, and 1 patient had an unexplained partial visual loss. The prognosis of vitrectomy correlated with TTR levels, suggesting the use of this protein as a marker of retinal function^(62)^.

### Open-angle glaucoma

Chronic open-angle glaucoma can result from conjunctival and episcleral perivascular amyloid deposits, elevated episcleral venous pressure, intratrabecular meshwork depositions, and deposits on the pupillary border^(31)^. Glaucoma was described already in early reports of ATTRv amyloidosis^(21,22)^. Its prevalence ranges from 5.4% to 27%^(33)^. It is a serious complication due to fast progression to visual loss and refractoriness to medical treatment. Kimura et al. reported secondary glaucoma in 25% of patients (mean age, 53.1+7.9 years) and a higher prevalence in non-Val30Met mutations^(27)^. Glaucoma was also associated with amyloid deposition (when seen on the pupil border, it marked the disease onset) and vitreous opacities^(27)^. It was also present in all cases of scalloped pupils^(27)^. As it can progress after liver transplant, mitomycin C-augmented trabeculotomy (0.4 mg/ml for 3-5 minutes intraoperatively) was reported as the preferred surgical treatment^(27)^. Selective or complete laser trabeculotomy was described as an alternative^(63)^. Complications of trabeculectomy included ocular decompression retinopathy (33%) and bleb encapsulation (48%); 57% of patients required an additional surgery (bleb revision or trabeculectomy)^(63)^. Glaucoma secondary to neovascular formation is discussed below.

### Vascular ophthalmological changes induced by altered TTR production

ATTRv amyloidosis can cause multiple vascular abnormalities of the eye, including the conjunctiva, retina, and choroid. These abnormalities (including amyloid deposits) have been described since the first publications on ATTRv amyloidosis but have not received widespread attention.

Ando et al. were probably the first to describe conjunctival microvascular angiopathy^(64)^. Abnormal conjunctival vessels (generally bilateral and punctiform) were found in 86.1% of the limbal area, with the exception of asymptomatic patients^(64)^. Amyloid deposits were identified in the superficial substantia propria of the conjunctiva as well as the wall and perivascular area of the conjunctival vessels^(65)^. Other series reported similar abnormalities in 61% to 75.7% of cases^(27,28)^. Conjunctival lymphangiectasia (i.e., distensions of the lymphatic vessels of the bulbar conjunctiva) was also described in 3 patients with ATTRv amyloidosis^(65)^. It developed as a result of scarring after trauma or surgery or due to Fabry disease^(65)^. They were reported in S77Y mutations with vessels showing a “string of pearls” appearance^(65)^.

Significant retinal disease is less common than conjunctival disease, but amyloid deposits around retinal and choroidal vessels are more widespread and can be present even in asymptomatic individuals^(24,28,33)^. Ando et al. also reported retinal vascular disease^(64)^, with cotton wool exudates in 8.7% and retinal hemorrhages in 26.1% of patients. In mutations with predominant cardiac involvement (E89K), retinal microangiopathy with retinal vasculitis leading to retinal ischemia and vitreous hemorrhage was described as the first manifestation^(66)^. Retinal vascular disease can also cause macular and optic disk edema, even in patients without vitreous involvement^(67)^. Indocyanine green angiography can also demonstrate evidence of occult choroidal vascular lesions, which can cause retinal hemorrhages and microaneurysms in patients with Val30Met and Y114C mutations^(68,69)^. Lastly, secondary glaucoma due to neovascularization or vascular changes is also a complication of vascular disease^(70,71)^. Rubeotic glaucoma following an uncomplicated vitrectomy for the treatment of vitreous amyloidosis was previously reported^(70)^. This patient had recurrent uveitis and developed glaucoma due to retinal neovascularization and extensive retinal vascular closure. Despite treatment with photocoagulation and Molteno implant surgery as well as intraocular pressure control, he developed vision loss. Another report described differences in the pathogenesis of glaucoma, in one case due to elevated episcleral venous pressure due to perivascular conjunctival amyloid deposits^(72)^.

### Ophthalmological complications in treated patients with ATTRv amyloidosis

Although most of TTR is formed in the liver, a smaller percentage is produced by the brain choroid plexus as well as ciliary and retinal pigment epithelium. It is not surprising that in the largest Val30Met series to date, the abnormalities in patients with and without liver transplant were similar^(26)^. Both vitreous amyloidosis and retinal amyloid angiopathy may continue to progress after transplant because they are both associated with mutant vitreous and cerebrospinal fluid TTR produced by the posterior retinal pigment epithelium^(26,51,61,73)^. In at least 1 patient with the Val30Met mutation who received liver transplant, macular and optic disk edema associated with retinal vascular leakage was identified and treated with panretinal anticoagulation as well as intravitreal injection of sustained-release dexamethasone^(67)^. Glaucoma progression, de novo glaucoma, amyloid deposits in the pupillary margin, as well as cardiac and leptomeningeal amyloid deposition have also been reported after liver transplant^(51)^. A few studies reported that tafamidis does not prevent progression of ocular disease^(74,75)^, although a trend for decreased severity was observed^(74)^.

### Guidelines for ophthalmological monitoring in ATTRv amyloidosis patients

Patients with ATTRv amyloidosis with and without liver transplant should undergo regular ophthalmological follow-up, because the long-term risk of severe eye involvement is high for both groups. Beirão et al. suggested the following visit schedule: 1) initial ophthalmological visit at the time of genetic diagnosis; 2) repeated evaluations every 2 years in asymptomatic carriers; and 3) annual evaluation in symptomatic patients^(26)^. Abnormal conjunctival vessels should be evaluated annually; lacrimal dysfunction, every 6 months after disease stabilization; and amyloid deposition in the iris or the anterior capsule of the lens, also every 6 months. Scalloped iris, glaucoma (after disease stabilization), vitreous amyloidosis (after surgery, if necessary), and retinal angiopathy (after laser therapy) should be evaluated every 3 months. *In vivo* confocal microscopy for imaging the corneal nerves may facilitate the concomitant evaluation of small fiber neuropathy and aid the management of patients with ATTRv amyloidosis^(76)^.

Eye involvement in ATTRv amyloidosis is a complex issue that requires an extensive knowledge of the different aspects of the disease course. A better understanding of ophthalmological complications is necessary, especially in Brazil where Portuguese ancestry is common. New therapies are warranted to prevent eye involvement and subsequent vision loss in patients with ATTRv amyloidosis.
